# *LEP* as a potential biomarker in prognosis of breast cancer: Systemic review and meta analyses (PRISMA)

**DOI:** 10.1097/MD.0000000000026896

**Published:** 2021-08-20

**Authors:** Tong Yi Jin, Madhuri Saindane, Kyoung Sik Park, SeongHoon Kim, SangEun Nam, YoungBum Yoo, Jung-Hyun Yang, IkJin Yun

**Affiliations:** a Department of Surgery, Konkuk University School of Medicine, Seoul, South Korea; b Research Institute of Medical Science, Konkuk University School of Medicine, Seoul, South Korea; c Department of Surgery, Konkuk University Medical Center, Seoul, South Korea.

**Keywords:** bioinformatics, breast cancer, leptin, obesity

## Abstract

**Purpose:**

Obesity strongly affects the prognosis of various malignancies, including breast cancer. Leptin (*LEP*) may be associated with obesity and breast cancer prognosis. The purpose of our study was to determine the prognostic value of *LEP* in breast cancer.

**Method:**

We conducted a multi-omic analysis to determine the prognostic role of *LEP*. Different public bioinformatics platforms (Oncomine, Gene Expression Profiling Interactive Analysis, University of California Santa Cruz Xena, bc-GenExMiner, PrognoScan database, R2-Kaplan–Meier Scanner, UALCAN, Search Tool for the Retrieval of Interacting Genes/Proteins database , and The Database for Annotation, Visualization and Integrated Discovery) were used to evaluate the roles of *LEP*. Clinicopathological variables were evaluated.

**Results:**

*LEP* was downregulated in breast cancer tissues compared to levels in normal tissues. By co-expressed gene analysis, a positive correlation between *LEP* and *SLC19A3* was observed. Based on the clinicopathological analysis, low *LEP* expression was associated with older age, higher stage, lymph node status, human epidermal growth factor receptor 2 (HER2) status, estrogen receptor (ER+) positivity, and progesterone receptor (PR+) positivity. Kaplan–Meier survival analysis showed that low *LEP* expression indicated a poorer prognosis. *LEP* is hypermethylated in breast cancer tissues in PrognoScan and R2-Kaplan Meier Scanner, and low *LEP* expression was correlated with poor prognosis. *LEP* protein–protein interactions were analyzed using Search Tool for the Retrieval of Interacting Genes/Proteins database. Gene ontology analysis results showed that cellular component is mainly associated with the endosome lumen, cytosol, and secretory granules and is upregulated. For the biological process energy reserve, metabolic processes exhibited the greatest regulation compared to the others. In molecular function, it was mainly enriched in a variety of combinations, but hormone activity showed the highest regulation.

**Conclusion:**

Our study provides evidence for the prognostic role of *LEP* in breast cancer and as a novel potential therapeutic target in such malignancies. Nevertheless, further validation is required.

## 1 Introduction

Breast cancer is one of the most common malignancies and the main cause of cancer-related deaths in women.^[1–4]^ Breast cancer is believed to involve genetic predisposition, but modifiable variables, such as overweight (body mass index 25–30 kg/m^2^) or obesity (body mass index >30 kg/m^2^), also play significant roles.^[5–7]^

Obesity is a growing public health problem that affects almost all countries.^[8]^ Obesity is regarded as a major risk factor for many severe illnesses, including diabetes mellitus, metabolic syndrome, cardiovascular diseases, and certain cancers.^[8,9]^ During the past decade, an explosion of evidence has linked obesity with an increase in the incidence of cancer and associated mortality.^[8,10]^ Obesity is also a major risk factor for breast cancer in postmenopausal women.^[8,11]^ Adipokines, which are bioactive molecules generated and secreted by adipocytes, may also contribute to postmenopausal breast cancer.^[12]^ Adipocytes produce nearly 50 different adipokines, which have various functions, such as regulation of inflammation and acute phase responses, insulin sensitivity, lipid metabolism, appetite, and energy balance.^[12,13]^ Because of its association with an increased risk of postmenopausal breast cancer, leptin (*LEP*) is the most studied adipokine^[12,14–19]^ and its role in promoting the growth and development of breast cancer in vitro has been recognized.^[12,20–25]^

*LEP* is directly associated with obesity. The circulatory rate of obese individuals is higher than that of lean individuals.^[12,26–28]^*LEP* can directly and independently promote the occurrence of breast cancer and indirectly promote the development of breast cancer through signaling pathways involving estrogen and insulin.^[12,29–31]^, which provides the basis for hormone-mediated carcinogenic interactions. Many studies have shown that *LEP* functions far beyond metabolism alone, and is the main mediator of obesity-related cancers, such as colorectal, prostate, and breast cancers.^[32,33]^

Herein, using public bioinformatics platforms, we evaluated the expression pattern of *LEP* in human breast cancer and the prognostic significance of *LEP* expression in breast cancer. Finally, we analyzed possible correlations involving genes co-expressed with *LEP*. These results would facilitate a better understanding of the prognostic value of *LEP* in human breast cancer.

## 2 Material and methods

### 2.1 Oncomine database analysis

The Oncomine database (https://www.oncomine.org/resource/login.html) is a web-based data-mining platform that incorporates 264 independent datasets and aims to collect, standardize, analyze, and deliver transcriptomic cancer data for biomedical research.^[34]^ The database was used to determine *LEP* expression levels in breast cancer. The fold change in *LEP* expression in clinical cancer specimens compared to normal controls was obtained using the parameters of *P* value 1e-4, fold-change 2, and gene ranking in the top 10%. The co-expression profile of the *LEP* gene in breast cancer is here displayed as a heat map.

### 2.2 GEPIA analysis

Gene expression profiling interactive analysis (GEPIA; http://gepia.cancer-pku.cn/) is a web server for analyzing the RNA sequencing expression data of 9736 tumors and 8587 normal samples from the The Cancer Genome Atlas (TCGA) and GTEx projects, using a standard processing pipeline. The GEPIA box-plot tool was used for tumor/normal differential expression analysis of *LEP* in different cancers.^[35]^

### 2.3 UCSC cancer genomics browser analysis

The heat map and correlation between *LEP* and *SLC19A3* in the same patient cohort were verified and analyzed by data mining in TCGA-BRCA using the University of California Santa Cruz Xena (UCSC Xena) browser (http://xena.ucsc.edu/). Subsequently, the methylation status of *LEP* was examined using the UCSC Xena browser.

### 2.4 Breast cancer gene expression miner v.4.2 analysis

Breast Cancer Gene-Expression Miner v.4.2 (bc-GenExMiner v.4.2) (http://bcgenex.centregauducheau.fr/BC-GEM/GEM-Accueil.php?js=1)^[36]^ was used to analyze the relationship between *LEP* expression and clinicopathological parameters in breast cancer patients.

### 2.5 PrognoScan analysis

PrognoScan (http://dna00.bio.kyutech.ac.jp/PrognoScan/) is an online platform used for meta-analysis of the prognostic value of genes.^[37]^ PrognoScan was used to assess the correlation between LEP expression and survival in breast cancer.

### 2.6 R2 platform analysis

R2 (https://hgserver1.amc.nl/cgi-bin/r2/main.cgi) is a genomics analysis and visualization platform with a database, coupled with a web interface that provides a set of analysis tools. R2 supports all types of survival data and can also be used to generate Kaplan–Meier plots for specific datasets.^[38]^ We analyzed correlations between *LEP* expression and survival in patients with breast cancer.

### 2.7 UALCAN analysis

UALCAN (http://ualcan.path.uab.edu/) is a website for online analysis and mining of cancer-related data. It is based mainly on the analysis of relevant cancer data from TCGA database. It can help perform biomarker identification, expression profiling, and survival analysis of related genes. It is possible to query related information from other databases through related links.

### 2.8 cBioPortal analysis

The cBioPortal for cancer genomics (http://www.cbioportal.org) is a widely used online portal that provides visualization and analysis of large-scale cancer genomics databases. In our study, cBioPortal was used to analyze the clinicopathological parameters of *LEP* in patients with breast cancer.

### 2.9 Identification of protein components of the *LEP* axis

We used the Search Tool for the Retrieval of Interacting Genes/Proteins database (STRINGdb) analysis tool (https://string-db.org/cgi/input.pl), a database of known and predicted protein interactions, to identify interacting proteins using *LEP* as the query.

### 2.10 GO analysis

GO analysis is a commonly used technique for large-scale functional enrichment research and can be classified into the biological process (BP), cellular component (CC), and molecular function (MF). To characterize the functional roles of the above proteins from STRING, the Database for Annotation, Visualization, and Integrated Discovery tools (https://david.ncifcrf.gov/home.jsp).

### 2.11 Statistical analysis

Overall survival (OS) was defined as the time from the first diagnosis of primary breast cancer to death from any cause. Survival curves were generated using PrognoScan and the R2-Kaplan–Meier plot. All results are displayed as *P* values using the log-rank test.

## 3 Results

### 3.1 *LEP* expression in human breast cancer

**Figure 1:**
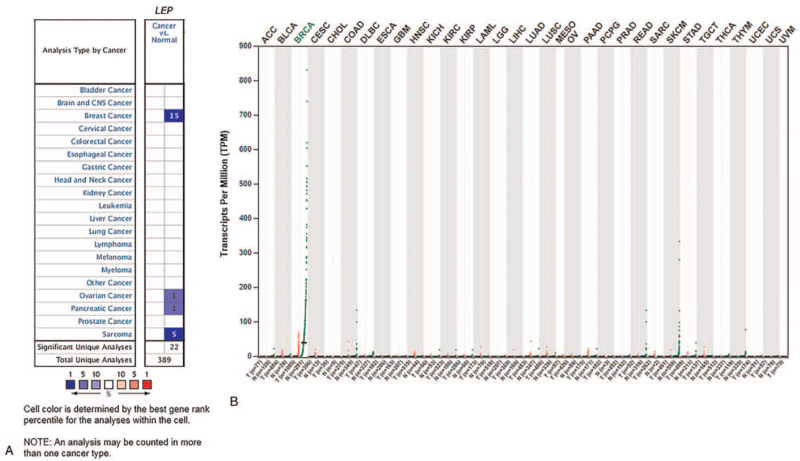
Expression of *LEP* in different types of cancers (Oncomine and TCGA databases). (A) This graphic generated by Oncomine indicates the numbers of datasets with statistically significant (*P* < .01) mRNA overexpression (red) or down-expression (blue) of *LEP* (different types of cancer vs corresponding normal tissue). The threshold was designed with the following parameters: *P* value of 1e-4, fold change of 2, and gene ranking of 10%. (B) Expression of *LEP* in different types of cancers, as generated by GEPIA using TCGA data. GEPIA = Gene Expression Profiling Interactive Analysis, LEP = leptin, TCGA = The Cancer Genome Atlas.

Oncomine analysis of cancer versus normal tissues showed that *LEP* was downregulated in breast cancer to a greater extent than in other cancers (Fig. 1A). *LEP* expression was significantly decreased in invasive breast carcinoma (–16.434), breast phyllodes tumor (−21.654), mucinous breast carcinoma (–15.065), breast carcinoma (–15.496), and medullary breast carcinoma (–15.482) (Table 1). GEPIA analysis showed that *LEP* expression in tumors was lower than that in normal tissues from the same breast cancer patients (Fig. 1B). Taken together, these results indicated that *LEP* is significantly downregulated in human breast cancer.

**Table 1:** The significant changes of *LEP* expression in transcription level between different types of breast cancer and normal breast tissues (Oncomine database).

Order	Type of breast bancer vs normal breast tissue	Fold change	*P* value	*t* test	Source
1	Invasive breast carcinoma	−16.434	7.67E-44	−20.133	Curtis breast
2	Breast phyllodes tumor	−21.654	1.94E-44	−23.768	Curtis breast
3	Mucinous breast carcinoma	−15.065	6.48E-45	−18.77	Curtis breast
4	Breast carcinoma	−15.496	4.58E-20	−15.711	Curtis breast
5	Medullary breast carcinoma	−15.482	5.93E-38	−17.9	Curtis breast
6	Ductal breast carcinoma in Situ	−11.683	7.58E-09	−10.338	Curtis breast
7	Tubular breast carcinoma	−11.709	1.22E-40	−16.702	Curtis breast
8	Invasive ductal and invasive lobular breast carcinoma	−12.448	4.41E-44	−17.467	Curtis breast
9	Invasive lobular breast carcinoma	−3.047	2.28E-45	−17.109	Curtis breast
10	Invasive ductal breast carcinoma	−3.201	8.87E-51	−21.993	Curtis breast
11	Invasive breast carcinoma	−3.184	1.09E-18	−12.46	Gluck breast
12	Mixed lobular and ductal breast carcinoma	−2.247	3.05E-09	−6.902	TCGA breast
13	Invasive ductal breast carcinoma	−10.754	4.06E-37	−18.577	TCGA breast
14	Male breast carcinoma	−2.024	6.27E-07	−6.055	TCGA breast
15	Invasive breast carcinoma	−4.619	2.08E-14	−8.481	TCGA breast

TCGA = The Cancer Genome Atlas.

### 3.2 Co-expression analysis of *LEP*

**Figure 2:**
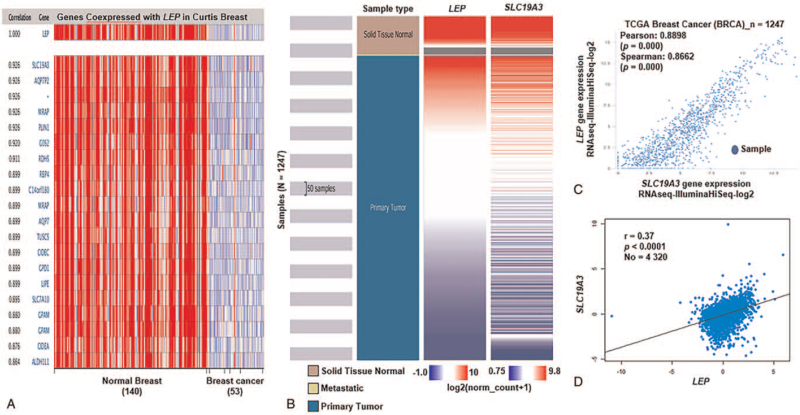
Co-expression analysis of *LEP* gene expression. (A) *LEP* co-expression of genes analyzed using Oncomine. (B) Heat map of *LEP* expression and *SLC19A3* mRNA expression across breast cancer sample types in TCGA database, determined using UCSC Xena. (C) Correlation between *LEP* and *SLC19A3* mRNA expression in TCGA database determined using UCSC Xena. (D) Relationship between *LEP* and *SLC19A3* in breast cancer analyzed using bc-GenExMiner v.4.2. LEP = leptin, TCGA = The Cancer Genome Atlas, UCSC XENA = University of California Santa Cruz Xena.

Oncomine analysis identified co-expression of *LEP* with a large cluster of 19,273 genes across 11 breast carcinomas (Fig. 2B). We confirmed this positive correlation for *SLC19A3* by analyzing breast cancer patient data in the TCGA database using UCSC Xena (Fig. 2B and C) and by data mining in bc-GenExMiner v.4.2 (Fig. 2D). These data indicated that *LEP* might be closely related to the SLCA3 signaling pathway in breast cancer.

### 3.3 Clinicopathological parameters of *LEP* in breast cancer patients

**Figure 3:**
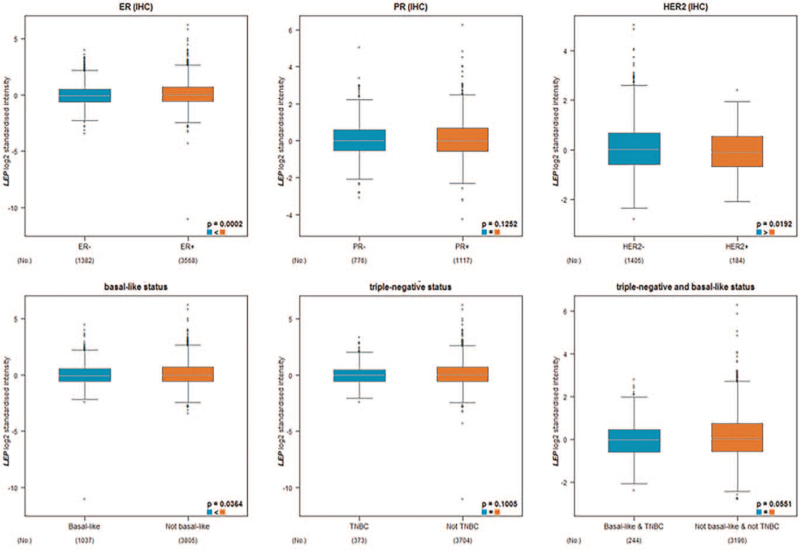
Relationship between *LEP* expression and clinicopathological parameters in breast cancer patients. The box plot was retrieved from bc-GenExMiner v.4.2. Global significant differences between groups were assessed by Welch *t* test to generate *P* values, along with Dunnett–Tukey–Kramer testing. LEP = leptin.

Among breast cancer patients, *LEP* expression was lower in the ≤51-year-old group than in the >51-year-old group (Table 2). *LEP* expression was significantly lower at the T1 and T2 stages than in the T3 and T4 groups. There was no significant difference in *LEP* expression between patients with positive and negative nodal status. According to staging, *LEP* expression was significantly lower in stages I, II, and III, but not in stage IV. Estrogen receptor (ER) status was positively correlated with *LEP* expression Conversely, *LEP* mRNA levels were lower in patients with human epidermal growth factor receptor 2 (HER2)-positive tumors than in those with HER2-negative tumors. *LEP* expression was significantly higher in basal-like status (Table 2). We performed Welch test, using bc-GenExMiner v.4.2, to compare *LEP* mRNA transcription levels among groups of patients according to different clinicopathological characteristics, and the above results were confirmed (Fig. 3). A significant correlation was found between low *LEP* expression and other clinicopathological variables.

**Table 2:** Clinicopathological relationship of *LEP* mRNA expression in breast cancer.

Variables	No.	*LEP* expression	*P* value	
Age	≤51	1312	Low	
	>51	2026	High	<.0001
T stage	T1	678	Low	<.0001
	T2	435	Low	<.0001
	T3	28	–	
	T4	13	–	
N stage	N0	2353	–	
	N1	1441	–	.0926
Stage	I	630	Low	<.0001
	II	979	Low	<.0001
	III	144	Low	<.0001
	IV	11	–	.0956
ER status	−	1382	Low	
	+	3568	High	.0002
PR status	−	776	–	
	+	1117	–	.1252
HER2 status	−	1405	High	
	+	184	Low	.0192
Basal-like status	−	3805	High	
	+	1037	Low	<.0364

*T* stage tumor stage, *N* stage node stage, *ER* status estrogen receptor, *PR* status progesterone receptor, *HER2* status human epidermal growth factor receptor 2.

### 3.4 Prognostic value of *LEP* mRNA expression in breast cancer patients

**Figure 4:**
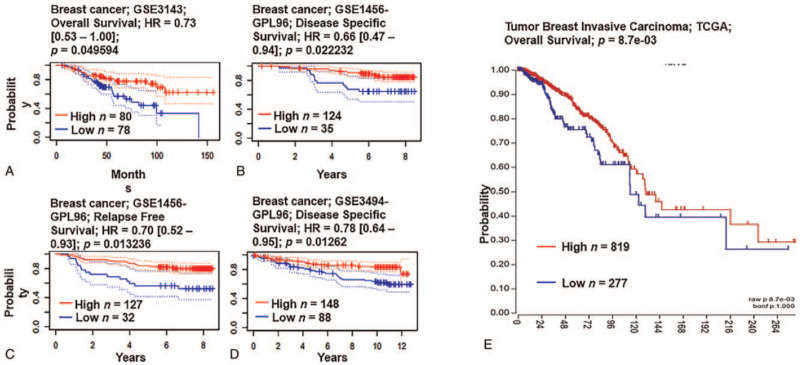
Relationship between *LEP* expression and clinicopathological parameters in breast cancer patients. Relationship between *LEP* mRNA expression and clinical outcomes in breast cancer patients (PrognoScan Database and R2: Kaplan–Meier Scanner). Relationship between *LEP* mRNA expression and clinical outcomes in breast cancer patients (PrognoScan Database and R2: Kaplan–Meier Scanner). (A–D). Survival curve comparing patients with high (red) and low (blue) expression of *LEP* was plotted from the PrognoScan database in breast cancer patients. Threshold of Cox *P* value <.05. (E) Survival curve comparing patients with high (red) and low (blue) expression of *LEP* was plotted from R2: Kaplan–Meier in TCGA breast cancer patients. The threshold of Cox *P* value <.05. LEP = leptin, TCGA = The Cancer Genome Atlas.

The prognostic value of *LEP* expression in breast cancer was explored using the PrognoScan database and R2: Kaplan–Meier Scanner. Higher expression of *LEP* correlated with better OS, relapse-free survival (RFS), and disease-specific survival (all *P* < .05) (Fig. 4). In our validation with the TCGA dataset, higher expression of LEP was correlated with better OS (*P* < .05).

### 3.5 Co-expressed genes, correlation, and DNA methylation status of *LEP*

**Figure 5:**
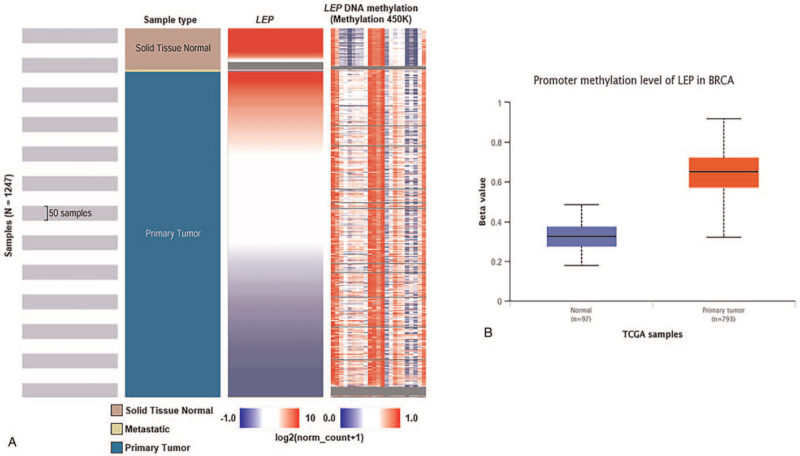
Correlation between *LEP* mRNA expression and promoter methylation in breast cancer (TCGA data). (a) Heat map of *LEP* expression and DNA methylation status retrieved from UCSC Xena. (b) Promoter methylation level of the *LEP* gene in breast cancer tissues compared to normal tissues. The box plot was retrieved from the UALCAN web server. LEP = leptin, TCGA = The Cancer Genome Atlas, UCSC XENA = University of California Santa Cruz Xena.

A correlation heat map and DNA methylation status retrieved from UCSC Xena confirmed that *LEP* expression gradually decreased with increasing DNA methylation (Fig. 5A). We also found that the methylation level of the *LEP* promoter was higher in breast cancer tissue than in normal tissue (Fig. 5B).

### 3.6 Protein–protein interactions

**Figure 6:**
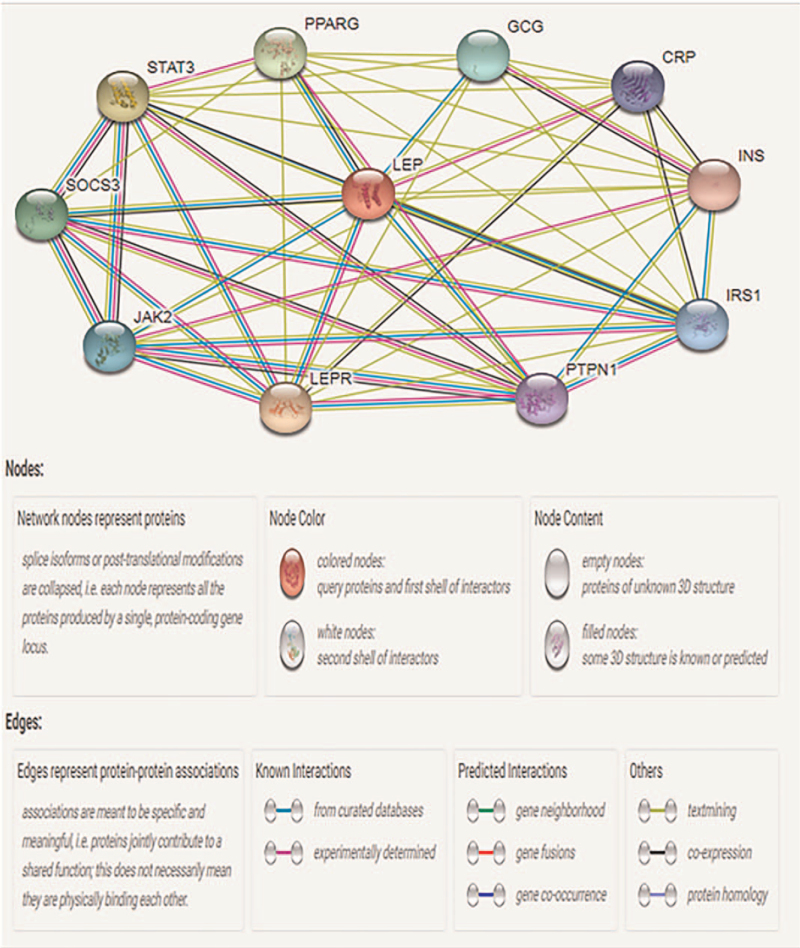
Identification of known and predicted structural proteins essential for *LEP* function (STRINGdb). LEP, STAT3, PPARG, GCG, CPR, INS, IRS1, PTPN1, LEPR JAK2, and SOCS3 interacting nodes are displayed as colored circles using STRING v.10.0. Predicted functional partners of *LEP* are shown based upon peer-reviewed published data and curated database entries. LEP = leptin.

Figure 6 illustrates the direct interactions and predicted functional associations involving *LEP* and other proteins retrieved from STRINGdb. The following proteins interacted with *LEP*: signal transducer and activator of transcription 3 (STAT3), peroxisome proliferator-activated receptor gamma (PPARG), glucagon (GCG), C-reactive protein (CRP), insulin (INS), insulin receptor substrate 1 (IRS1), tyrosine-protein phosphatase non-receptor type 1 (PTPN1), leptin receptor (LEPR), tyrosine-protein kinase JAK2 (JAK2), and suppressor of cytokine signaling 3 (SOCS3).

### 3.7 GO analysis

**Figure 7:**
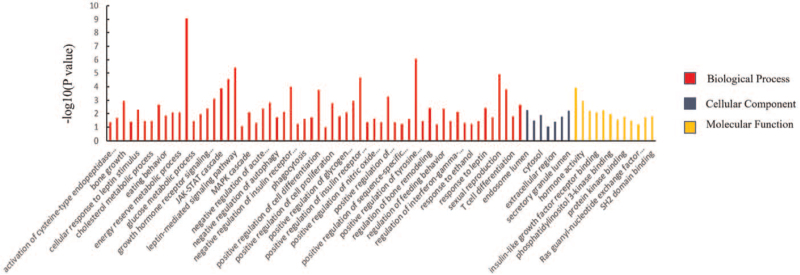
GO analysis using DAVID tools. DAVID = The Database for Annotation, Visualization and Integrated Discovery, GO = gene ontology.

The above proteins were classified into the 3 functional groups of GO analysis: CC, BP, and MF (Fig. 7). Numerous BPs were identified (Fig. 7). The results of the GO analysis showed that CC is mainly associated with the endosome lumen, cytosol, and secretory granule lumen and is upregulated. For the BP energy reserve, metabolic processes have the highest regulation compared to the others. In MF, it was mainly enriched in a variety of combinations, but hormone activity showed the greatest regulation.

## 4 Discussion

In the present study, we examined the prognostic significance of *LEP* in breast cancer. *LEP* is considered a potential breast cancer susceptibility gene.^[15]^ Most previous studies have shown an association between high *LEP* expression levels and poor prognosis in several cancers.^[8,39]^ In contrast to these previous studies, low levels of *LEP* were seen to play an unfavorable role in breast cancer progression in our data analysis. Serum *LEP* abundance is higher in patients with breast cancer than in controls, and higher levels of serum *LEP*, intratumoral *LEP* mRNA, and intratumoral *ObR* isoform mRNA levels have been observed to be poor prognostic predictors in patients with breast cancer.^[8,16,40]^ In this study, we first analyzed the expression of *LEP* in breast cancer tissues and other malignancies and found that *LEP* expression in breast cancer was downregulated compared to that in other tumor types. We validated these results by analyzing the data surrounding *LEP* expression in different types of cancers generated by GEPIA using TCGA data (Fig. 1). An assessment of the comprehensive median expression of *LEP* across 15 analyses using the *Curtis Breast* microarray dataset showed that *LEP* expression was downregulated in most breast cancer types (Table 1).

Our results contradict previous research that indicates the involvement of serum *LEP* in the progression of established breast tumors. However, it also speculates that a high serum *LEP* level may be associated with breast carcinogenesis since *LEP* stimulates the proliferation of normal human breast epithelial cells in vitro.^[32]^ The difference in study results could be the following reasons. Firstly, serum *LEP* levels in breast cancer patients and healthy women are not significantly different. Second, serum *LEP* levels do not show a significant difference between tumor stages. Third, *LEP* mRNA levels in tumor tissue are much lower (1/12th) than those in adipose tissue (data not shown). Thus, the reason for the association between serum *LEP* levels and *LEP* mRNA levels is different and hence the contradiction.^[32]^

In this study, through co-expression analysis, we found that *LEP* expression was positively correlated with *SLC19A3* expression. Previous research suggests that *SLC19A3* is downregulated in breast cancer by hypermethylation of a CpG-rich region in its promoter.^[41,42]^ The positive correlation between *SLC19A3* and *LEP* expression in our study indicates its role in breast cancer progression and metastasis (Fig. 2).

The main role of *LEP* is in modulating ER signaling and aromatase activity.^[43,44]^ Increased expression of *LEP* and *LEP* receptors in both primary and metastatic breast cancer tissues and correlation of the expression of these proteins with ER status, tumor size, and higher tumor grade have been documented in several studies.^[8,45]^*LEP* and estrogen may cooperate in maintaining estrogen-dependent breast cancer growth.^[8]^*LEP* can increase aromatase activity, promote the production of estrogen, and thus stimulate the development of ER^+^ breast cancer.^[46]^ In the current study, ER status was positively correlated with *LEP* expression. Conversely, basal-like and HER2-positive subtypes were negatively correlated with *LEP* expression. Nevertheless, more data are required to elucidate the mechanisms underlying *LEP* expression and HER2 status. The evolution of estrogen-dependent breast cancer is primarily caused by ER signals that can be activated by *LEP* signaling,^[46]^ which may explain the positive association between ER^+^ breast cancer patients in the current study and breast cancer recurrence (Table 2, Fig. 3).

One study reported that high expression of *Ob-R* mRNA in breast cancer tissue indicates a poor prognosis for patients with high serum *LEP* levels.^[47]^ A previous study reported that a shorter RFS duration was associated with mRNA expression of the short isoform of the *LEP* receptor (OB-R-S).^[47]^ In contrast, our results showed that low *LEP* expression correlated with shorter OS, RFS, DMFS, and disease-specific survival, and Kaplan–Meier survival analysis indicated that *LEP* expression was associated with reduced OS in breast carcinoma patients, indicating that low expression of *LEP* may be functionally important in tumor progression (Fig. 4).

In addition, DNA methylation is an important regulator of gene transcription, and such epigenetic modifications are common in many tumors and during development. Global hypomethylation is considered a cause of cancer.^[48]^ In our analysis, comparing the *LEP* expression heatmap and DNA methylation status revealed that *LEP* expression gradually decreased with increasing DNA methylation, indicating that *LEP* methylation levels were higher in breast cancer tissue than in normal tissue from the same patients (Fig. 5).

Finally, we analyzed the relationships between *LEP* and interacting proteins using STRINGdb. We found that STAT3, PPARG, GCG, CRP, INS, IRS1, PTPN1, LEPR, JAK2, and SOCS3 directly interact with *LEP* (Fig. 6). Using these proteins, we performed GO functional annotation analysis using The Database for Annotation, Visualization and Integrated Discovery. GO analysis, the establishment of the PPI network, and network topological analysis were utilized to identify genes potentially related to the initiation and development of *LEP* (Fig. 7). GO functional analysis indicated that *LEP* was enriched in hormone activity for MFs, indicating its role as a hormone.^[49]^

In summary, *LEP* may play a role in breast carcinoma development. Nevertheless, there is a lack of in-depth insight into the relevant mechanisms involved, and further research is required. It will also be necessary to determine the normal reference range for *LEP* levels in the healthy population. Further longitudinal studies are required to explain the relationship between *LEP* expression levels and the risk of breast cancer. We believe that new medicines could be created to combat breast cancer and other breast diseases with further studies involving *LEP*. Overall, our study suggested that *LEP* can be used as a positive prognostic biomarker and a potential therapeutic target in breast cancer.

## Acknowledgments

None.

## Author contributions

TYJ, MS, and KSP conceived the analysis and wrote the manuscript. SEN, YBY, JHY, IJY, and SHK conducted proofreading. All authors have reviewed the manuscript.

**Conceptualization:** Madhuri Saindane.

**Data curation:** Tong Yi Jin.

**Formal analysis:** Tong Yi Jin, Kyoung Sik Park, Jung-Hyun Yang.

**Funding acquisition:** Kyoung Sik Park.

**Investigation:** SeongHoon Kim, YoungBum Yoo.

**Methodology:** SangEun Nam.

**Project administration:** Kyoung Sik Park.

**Resources:** Kyoung Sik Park.

**Software:** Madhuri Saindane.

**Validation:** Kyoung Sik Park, SeongHoon Kim, SangEun Nam, IkJin Yun.

**Visualization:** Madhuri Saindane.

**Writing – original draft:** Tong Yi Jin.

**Writing – review & editing:** Tong Yi Jin, Kyoung Sik Park.

## Correction

When originally published, the acknowledgement that “This work was supported by Konkuk University Medical Center Research Grant in 2020” was omitted. This has been added to the footnote on the first page.
